# Defining the Digital Measurement of Scratching During Sleep or Nocturnal Scratching: Review of the Literature

**DOI:** 10.2196/43617

**Published:** 2023-04-18

**Authors:** Will Ke Wang, Lucia Cesnakova, Jennifer C Goldsack, Jessilyn Dunn

**Affiliations:** 1 Department of Biomedical Engineering Duke University Durham, NC United States; 2 The Digital Medicine Society Boston, MA United States; 3 Department of Biostatistics & Bioinformatics Duke University Durham, NC United States; 4 The Duke Clinical Research Institute Durham, NC United States

**Keywords:** atopic dermatitis, ontologies, nocturnal scratching, quality of life, outcomes measurement, dermatitis, scratch, review method, systematic review, literature review, pruritis

## Abstract

**Background:**

Digital sensing solutions represent a convenient, objective, relatively inexpensive method that could be leveraged for assessing symptoms of various health conditions. Recent progress in the capabilities of digital sensing products has targeted the measurement of scratching during sleep, traditionally referred to as nocturnal scratching, in patients with atopic dermatitis or other skin conditions. Many solutions measuring nocturnal scratch have been developed; however, a lack of efforts toward standardization of the measure’s definition and contextualization of scratching during sleep hampers the ability to compare different technologies for this purpose.

**Objective:**

We aimed to address this gap and bring forth unified measurement definitions for nocturnal scratch.

**Methods:**

We performed a narrative literature review of definitions of scratching in patients with skin inflammation and a targeted literature review of sleep in the context of the period during which such scratching occurred. Both searches were limited to English language studies in humans. The extracted data were synthesized into themes based on study characteristics: scratch as a behavior, other characterization of the scratching movement, and measurement parameters for both scratch and sleep. We then developed ontologies for the digital measurement of sleep scratching.

**Results:**

In all, 29 studies defined inflammation-related scratching between 1996 and 2021. When cross-referenced with the results of search terms describing the sleep period, only 2 of these scratch-related papers also described sleep-related variables. From these search results, we developed an evidence-based and patient-centric definition of nocturnal scratch: an action of rhythmic and repetitive skin contact movement performed during a delimited time period of intended and actual sleep that is not restricted to any specific time of the day or night. Based on the measurement properties identified in the searches, we developed ontologies of relevant concepts that can be used as a starting point to develop standardized outcome measures of scratching during sleep in patients with inflammatory skin conditions.

**Conclusions:**

This work is intended to serve as a foundation for the future development of unified and well-described digital health technologies measuring nocturnal scratching and should enable better communication and sharing of results between various stakeholders taking part in research in atopic dermatitis and other inflammatory skin conditions.

## Introduction

Atopic dermatitis (AD) is a chronic inflammatory disease of the skin that affects up to 25% of children [1] and about 10% of adults in high-income countries [2]. Its most common and bothersome symptom is itch (pruritus), which is an unpleasant skin sensation. Pruritus can result in an urge or reflex to scratch, which further damages the skin and results in a continuous itch-scratch cycle [3], which can in turn have major deleterious effects on patients’ quality of life and clinical course. Moreover, the terms itch and scratch are often used interchangeably, not distinguishing between the sensation of itch and the action of scratching.

The need to assess the severity of AD symptoms, including itch and scratching, both in clinical practice and in clinical trials of therapeutics is widely recognized by researchers and clinicians. Survey-based or observational measurement of scratching is strongly related to the broader assessment of the subjective pain-like [4] sensation of itch [5]. Scratching, particularly during times of rest that usually happen at night, can result in disturbed sleep for both patients [6-8] and others in the household [9]. During periods of rest and sleep, the willful regulation of behaviors subsides, and other regular daily movements do not interfere with scratching movements. That is why nocturnal scratching has been recognized as a promising novel measurement [10] that brings new insight complementary to current AD measures, offering an opportunity for continuous measurements out of the clinic but also with a certain degree of objectivity. Digital health technologies (DHTs) allow researchers of nocturnal scratch and AD to collect and assess quantifiable information about the behavior of scratching and its impact on sleep and, hence, enhancing our toolbox of assessments to uncover complex relationships between itch, scratching, and sleep, as well as the changes induced by treatments [11].

No definitive laboratory test or outcome assessment exists to diagnose or track AD continuously, but several clinical outcome assessments—whether clinician or patient reported, or their combination—have been validated for assessing AD severity [12]. One of the most commonly used is the Scoring Atopic Dermatitis Index (SCORAD) [13,14], which classifies AD severity according to the body area affected and the characteristics of the skin lesions. Other established assessments include the Investigator Global Assessment Scale (IGA) [15]; the Eczema Area and Severity Index (EASI) [16]; and the Six Area, Six Sign Atopic Dermatitis Scale (SASSAD) [17]. However, these assessments have several limitations, as they are subject to substantial interobserver variability [18], are unable to capture the full itch-scratch cycle (and its fluctuations), and are administered in the clinic. From the patient’s perspective, the Patient-Oriented Eczema Measure (POEM) [19,20] captures the frequency of itching and disturbed sleep, but it is subjective; can be influenced by mood or suggestion; is subject to noncompliance; and does not capture the duration, severity, or timing of scratching. Only a few of these survey and questionnaire tools look deeper into the intensity and impacts (including sleep related) of itch and scratch [21], while direct, semiobjective, and scalable methods are missing. The current gold standard method based on direct observation of nocturnal scratching and sleep in a single patient—videography in a sleep laboratory assessed by a trained rater—is impractical to scale, is costly, and poses challenges to longitudinal monitoring of disease activity and effects on sleep. An objective convenient measurement of nocturnal scratching would be of great value to assess its impact on daily life, monitor fluctuations in the disease course [22], and indicate therapeutic efficacy in trials.

Continued advances in DHTs and machine learning algorithms show promise in the measurement of both nocturnal scratching and sleep parameters [23]. They offer the advantages of being able to capture continuous objective data inexpensively, in real time, and in patient populations that cannot report their itch or scratching by themselves [24]. Some drawbacks of these methods are the possibility of missing low-grade scratching and mislabeling sleep versus wake epochs [25]. We found that aggregating or comparing findings across studies in this realm are hampered by variations in definitions for both the “nocturnal” period (or “sleep”), during which nocturnal scratching occurs, and “scratching” itself. We conducted a narrative review of the literature looking for definitions used to date. Based on our findings, we developed unifying evidence-based definitions for “nocturnal scratch,” specifically in the context of digital measurement of scratching during sleep periods and being distinct from describing the sensation of itch. In this paper, we propose ontologies—a set of concepts, their properties, and the relations between them—to advance the standardization of definitions and outcome measurements for the digital measurement of nocturnal scratching.

## Methods

### Overview

**Figure 1:**
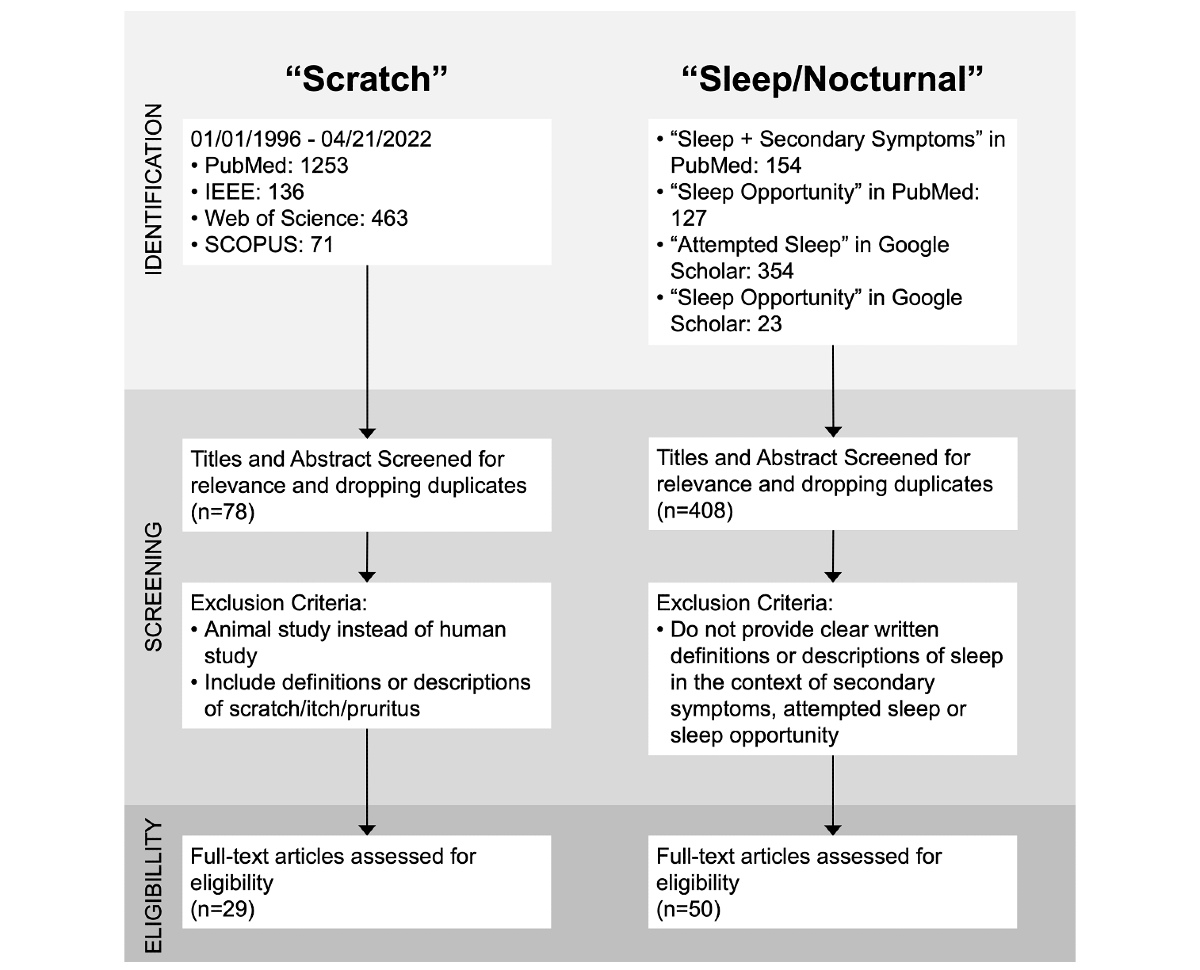
Literature search and screening pipeline.

We performed a narrative literature review of definitions of scratching in patients with skin inflammation and a targeted literature review of sleep in the context of the period during which such scratching occurred. Then we synthesized these findings to propose an evidence-based definition of the novel measure, nocturnal scratch. This process is illustrated in Figure 1. A formal protocol was not registered prospectively.

### Narrative Review of Scratch Definitions

#### Search Strategies

For the “scratch” search, from March to April 2022, we searched for English-language studies published between January 1996 and March 2022 in the PubMed, IEEE, Scopus, and Web of Science databases, limiting the search to dermatitis-related studies in humans. We decided to narrow the scope of the searches to include only papers or articles that had 1 or more of these terms in the title to ensure their relevancy and that they discussed or examined scratch or related topics. This approach was selected after evaluating a preliminary search for the terms “scratch,” “itch,” or “pruritus” in the full text. This approach returned too many papers, the majority of which did not discuss scratch or itch to any degree of importance. The specific search strings used are shown in Table S1 in Multimedia Appendix 1 [11,25-52].

#### Inclusion and Exclusion Criteria

For the narrative review of scratch definitions, the titles were screened for the presence of 1 of the search terms in the title as described above, and then 1 of the authors (WKW) and an assistant (see the Acknowledgments) searched the full-text versions of retrieved publications for the presence of any definition of scratching. Studies were excluded if they did not target human research (eg, animal studies). Papers were also excluded if they did not contain both a search term in the title and a definition for scratching. No restrictions were placed on study design, sample size, age of the cohort, or publication type.

#### Data Extraction

One author (WKW) and an assistant independently extracted the following scratch-related data from each included study: author, year published, cohort size, definition of scratching behavior, tool used for scratching, scratching sites assessed, definition for scratch duration, clinical outcome or calculated index used, device used to capture scratching, sleep definition or terminology used, and sleep detection method. Differences between extractors were resolved by consensus. These data were extracted qualitatively and tabulated by year using Word (Microsoft Corporation). Two of the other authors (LC and JD), along with an assistant, reviewed all of the tabular data to ensure that no definitions or descriptions of interest were missed or misrepresented.

### Targeted Review of Sleep Definitions

#### Search Strategies

Compared with scratching, sleep has a much wider and more systematic literature of research. We recognized that the definitions of sleep and its different stages and characterizations are much better described. For the purpose of identifying relevant articles targeting events, symptoms, or conditions happening during sleep, we selected 3 search strategies: searching for sleep-related symptoms and conditions (cramp, pain, spasm, etc) together with a term for the sleep period (sleep, rest, nap, nocturnal, etc), the specific term “sleep opportunity,” and the specific term “attempted sleep” (Table S2 in Multimedia Appendix 1). Similarly to the scratch search, we limited the search to titles only to ensure the relevancy of the results, apart from 1 of the search terms that targeted a lesser-used term (“attempted sleep” in all fields of Google Scholar).

We performed the searches from March to April 2022. We limited the search for sleep-related symptoms and conditions to PubMed only, as we wanted to target specifically journal articles to look for clinical evidence. We deemed PubMed suitable for this cause, as Google Scholar is known to also include books, scholarly websites, or conference proceeding records. For the term “sleep opportunity,” we performed a title search in both the PubMed and Google Scholar databases. For the term “attempted sleep,” we performed a title search in PubMed and an all-field search in Google Scholar. The specific search strings are shown in Table S2 in Multimedia Appendix 1.

#### Inclusion and Exclusion Criteria

For the targeted review of sleep definitions, we screened the abstracts for the presence of a definition or description of sleep in the context of secondary symptoms for a researched disease, as well as a definition or description of sleep opportunity or attempted sleep. Studies were excluded if they did not contain the definition of sleep period or if sleep was not evaluated in the context of the disease that might have effects on sleep. No restrictions were placed on study design, sample size, age of the cohort, or publication type.

#### Data Extraction

For sleep, the same individuals extracted the author, year published, cohort size, sleep definition or terminology used, and sleep detection method. These data were extracted qualitatively and tabulated by year using Word. Two of the other authors (LC and JD), along with an assistant, reviewed all of the tabular data to ensure that no definitions or descriptions of interest were missed or misrepresented. As the literature on the topic is already robust and well rounded, instead of reporting on each article in detail, we elected to incorporate the findings directly into the sleep ontology as a result of this work.

### Synthesis and Development of Proposed Definitions for Broad Adoption

The extracted data were synthesized into themes based on study characteristics: scratch as a behavior, other characterization of the scratching movement, and measurement parameters for both scratch and sleep. Because of the heterogeneities in study designs, outcome measures, and reporting of outcomes, meta-analytic techniques could not be used to further summarize the studies.

The synthesis of the results was performed with 2 goals: (1) to identify the most fitting and contextually correct definitions for “scratch” and “nocturnal” (in the context of sleep) and (2) to identify the parameters of the measurement of “scratch” and “nocturnal” to inform the development of ontologies for the novel measurement.

To achieve the first goal, we reviewed all the findings from the literature search with members of a multistakeholder project team composed of experts in AD, sleep, technology development, drug development, clinical care, and the patient and care partner experience (see the Acknowledgments), and ranked the metrics based on clinical and patient relevance.

For the development of ontologies for the novel measurement of nocturnal scratch, we adopted the contextual definitions from the first step to serve as a main class under which the related concepts and properties were nested. We evaluated the properties of our selected measures of scratch and sleep and then organized them under the core concepts connected to the definitions. These core concepts represent both the specific subparts of the measure of interest and the technical features of each measurement to be reported when assessing nocturnal scratch. To increase understanding and promote adoption, we added example values (or nested properties, where applicable) that illustrate the information the specific properties add to the overall measurement.

Finally, we reviewed these findings with regulatory colleagues from the US Food and Drug Administration during a Critical Path Innovation Meeting on July 22, 2022 [53].

## Results

### Narrative Review of Scratch Definitions

We decided to apply a 25-year time constraint on our literature search (ie, between 1996 and 2021) to capture the most recent and up-to-date definitions or descriptions of “scratch.” We purposefully started the search in the 1990s, the era of the proliferation and maturing of new accelerometry-based devices measuring movement and activity [54]. From 1996 to 2021, a total of 1253 unique publications with at least 1 of the search terms in their titles appeared in PubMed. Additional publications were found in the searches of IEEE (n=136), Scopus (n=71), and Web of Science (n=463). After removing duplicates and applying the inclusion/exclusion criteria to all found articles, 29 studies contained a definition of scratch, itch, or pruritus. The terms “itch” and “pruritus” were added due to the frequently interchanged use of the terms itch and scratch. A summary of these studies is presented in Table 1 [11,25-52], and their full details are included in Table S3 in Multimedia Appendix 1.

**Table 1:** Summary characteristics of studies included in the review.

Author and year	Study participants, n	Clinical outcome or calculated index	Device	Scratch definition/terminology	Scratch detection method
Ebata et al [26], 1996	10	TST^a^, light rubbing (total duration + count), long bouts of scratching (total duration + count), short bouts of scratching (total duration + count)	Infrared camera	Some patterns of scratching were observed and then classified into 3 categories: (1) light rubbing, touching, or scratching lasting <5 s; (2) short bouts of scratching with a duration 5-30 s; and (3) long bouts of scratching with a duration >30 s.	Manual recognition/count through video recording
Endo et al [27], 1997	43	Scratch rate, minute scratch records	“Scratch monitor” (accelerometric device)	N/A^b^	Pressure changes on scratch monitor
Ebata et al [28], 1999	35	TST	Infrared camera	An apparent action of rubbing or scratching to any part of the body with a rhythmical movement using hands or fingers and sometimes feet lasting >5 s.	Manual recognition/count through video recording
Endo et al [29], 1999	40	Scratch rate, minute scratch records	“Scratch monitor” (accelerometric device)	N/A	Pressure changes on scratch monitor
Ebata et al [30], 2001	55	TST (at night)	ActiTrac monitor	An apparent action of rubbing or scratching to any part of the body with a rhythmical movement using hands or fingers and sometimes feet lasting >5 s.	Piezoceramic sensor calibrated to units of acceleration
Munday et al [31], 2002	155	Excoriation score	Innovaderm digital visual analog scale device	N/A	N/A
Benjamin et al [32], 2004	21	Proportion of total measured time (%) spent in 1 of the following states: sleep, scratching, restless movement, movement under covers	Piezoelectric accelerometer, video recording	Scratching was defined to include rhythmic movements, judged by eye, >1 Hz without accompanying movements. By contrast, restlessness, such as frequent turning over in bed, slow rubbing of the face on the pillow, or writhing movements of the limbs, could include short periods of scratch (1 or 2 s) as long as these latter movements were interspersed with other nonscratch activities. Therefore, whereas scratching activity (in the absence of restlessness) could be usefully demarcated, short periods of scratching interspersed with restlessness could not be separated and were summarized as restlessness. Movement under the covers refers to obvious movement that could not be assessed definitely as either belonging to restlessness or scratching.	Piezoelectric accelerometer
Hon et al [33], 2006	39	Wrist activity	DigiTrac monitor	Wrist activities 1-3 Hz	N/A
Hon et al [34], 2007	28	Wrist activity for first 3 h of sleep	DigiTrac monitor on dominant wrist	Limb motion 1-3 Hz	N/A
Ishiuji et al [35], 2008	26	Scratch performed by electronic device	Medi-Pak cytology brush	Willful scratching: Scratching was accomplished by study personnel by repetitively moving a cytology brush over the ventral forearm 3 cm distal to the iontophoresis site.	N/A
Bender et al [36], 2008	20	Scratching index (not further defined)	Polysomnography, electromyograph	A scratching event was recorded as such when a burst of electromyographic activity of ≥3 s was accompanied by a visible scratching motion.	Electromyographic activity accompanied by visible scratching motion
Tran et al [37], 2010	45	Itch score (0-10)	Medi-Pak cytology brush	Willful scratching: Participants underwent artificial scratching over the area of the forearm 3 cm distal to the edge of the area of histamine iontophoresis for 2 min. Scratching was accomplished by study personnel repetitively moving a cytology brush over the ventral forearm.	N/A
Petersen et al [38], 2013	12	Activity over first 3 h of sleep; number of accelerations >0.01 *g*	Accelerometer, acoustic, and others	From referenced papers: movement with frequency of 1-2 Hz	Triaxial accelerometry + MLM^c^ of the signal data (logistic regression model)
Kurihara et al [39], 2013	10	TST percentage; clinician visual estimate of scratching time	Electromyography, microphone, accelerometer, triaxial gyro sensor	Willful scratching: The participant moves his right hand to his right cheek and uses his fingers to scratch his cheek 20 times and then returns to the starting position with his arm at his side on the bed. Scratching 1 time was 1 scratching stroke.	Changes in myogenic potential of forearm, pressure changes on back of hand, sounds from scratching, expansion/contraction of fingers, acceleration/angular velocity of forearm
Price and Cohen [11], 2014	N/A	TST/percentage of recording, SCORAD^d^ score	Infrared video, actigraphy, acoustic device	An apparent action of rubbing or scratching to any part of the body with a rhythmical movement using hands or fingers and sometimes feet lasting >5 s.	Manual recognition/count through video recording
Yamanaka et al [40], 2015	20	TST percentage	Wristwatch, electroencephalogram	N/A	Wristwatch-type scratching counting system detecting small scratching sounds from fingertip at wrist
Schut et al [41], 2015	48	Scratching induced by experimental video vs control video	Video and questionnaire	Any movement that included rubbing (mere touching did not count as scratching)	N/A
Lee et al [42], 2015	3	Number of times of scratching	Accelerometer	The distance moved, the velocity of the wrist, and the number of scratchings. Because scratching is a periodic movement, consecutive changes of direction of the wrist movements were regarded as the number of scratchings. People may use their wrists and fingers to scratch, but often they use only their fingers. Although scratching with the fingers without significantly moving the wrist causes a tiny amount of acceleration, the acceleration pattern produces the same graph as the pattern of scratching with wrist movement.	Accelerometry
Kurihara et al [43], 2015	NR^e^	Degree of finger bending (to reflect scratching motion)	Piezoceramic sensor between 2 metal plates	Willful scratching: The participant moves his right hand to his right cheek and uses his fingers to scratch his cheek 20 times and then returns to the starting position with his arm at his side on the bed. Scratching 1 time was 1 scratching stroke.	Amplitude of vibrations produced by scratching
Camfferman et al [44], 2016	33	SCORAD scratch	Polysomnography, video	Recording of scratch movements by the participant using their hand, leg, etc. Video recorded in conjunction with overnight polysomnography later reviewed by a sleep technician for scratching events. The duration of all scratching events was measured.	Manual recognition/count through video recording
Kaburagi and Kurihara [45], 2017	12	TST percentage, number of scratching periods	Accelerometer, gyro sensor	Willful scratching: The participant scratches his right cheek 20 times using the fingers of his right hand. He then brings his arm back to the starting position. After a break of 5 s, the participant once again scratches his cheek 20 times in a row. This set of scratching motion is repeated 35 times.	Degree of finger bending, acceleration and angular velocity of forearm
Bartels et al [46], 2018	97	Frequency of localized scratching (primary outcome), frequency of total body scratching (secondary outcome), duration of localized and total body scratching (exploratory)	Video camera	Any skin contact movement that could reduce itch (eg, typical scratching using the fingernails, picking with fingernails, or rubbing) while not taking into account touching.	Manual recognition/count through video recording
Moreau et al [47], 2018	24	TST	Wrist actigraphy infrared camera	An apparent action of rubbing or scratching to any part of the body with a rhythmical movement using hands or fingers.	2 accelerometer devices, video + MLM of high-resolution actigraphy signals using bidirectional recurrent neural network
Sanders et al [48], 2019	N/A	N/A	N/A	Neurological: Scratching activates spinal interneurons that inhibit itch-sensitive neurons, suppressing the transmission of itch signals to the brain. Scratching an itch also deactivates the amygdala, calming our negative emotions. In this rare case, pain becomes something that we actually seek out to experience relief from itch. The pleasure of scratching is correlated with activity in major reward centers of the brain, such as the ventral tegmental area and the nucleus accumbens. Therefore, in scratching an itch, we experience 2 rewards: relief of the itch (negative reinforcement) and pleasure (positive reinforcement).	N/A
Ikoma et al [49], 2019	236	TST percentage of total sleep duration	Video, accelerometer in Apple smart watch	This study predefined an acceleration pattern that their algorithm is looking for. Each participant wore 2 identical smart watches (Apple Watch Sport 38 mm, Apple Inc, United States) in which the app was installed, with 1 watch on each wrist. The evaluator, who was blinded to the smart watch data, watched the video of the participants and recorded the timing and duration of all body motions during sleep. If the motions were judged as scratching by the evaluator, they were recorded together with the information on which arm was used and categorized into 3 grades depending on the intensity of scratching motions; “weak” if only fingers were used to scratch without visible movement of the elbow, “moderate” if the elbow visibly moved, and “strong” if both the elbow and the shoulder visibly moved.	Changes in acceleration data with algorithm analysis
Kamath et al [50], 2020	37	Clinician Scratch Scale	Diary/questionnaire	N/A	Visual observation
Mahadevan et al [25], 2021	33	TST, number of events	Wrist accelerometer/ambient light sensor/temperature sensor; thermal videography	Any repetitive rubbing or scratching of the body (including through fabric) performed with the hand or any part of the upper limb (from [30]). Initiation frame: First contact of any part of the hand—including fingers, nails, palm, or dorsum—with the scratch area, continuous with the scratching behavior. Termination frame: Last contact of any part of the hand—including fingers, nails, palm, or dorsum—with the scratch area, or the last visible movement of the hand if there is a ≥3-s pause in scratching with the hand still in contact with the scratching area.	Visual observation, device outputs + established sleep detection algorithm and MLM of the actigraphy data using feature engineering and random forest classifier
Hong et al [51], 2021	399	Survey scores	Surveys	Severity of scratching/excoriation (SCORAD scratch)	N/A
Chun et al [52], 2021	21	TST	Infrared video, acousto-mechanic device strapped to Apple Watch 4	Scratching can occur via articulation of the elbow, wrist, or fingers alone. In the mode that engages the entire upper arm, the fingers press against the surface and remain stationary, where movement of the forearm articulating at the elbow joint drives the scratching action. The finger scratching mode, on the other hand, involves mainly local movements of the fingers, without substantial forearm or wrist motion. Mixtures of these 2 modes are also possible. Depending on the location of scratching and the degree of itch, individuals interchangeably use different modes of scratching.	Acousto-mechanic signals, visual observation + MLM of accelerometer data using feature engineering and random forest classifier

^a^TST: total scratch time.

^b^N/A: not applicable.

^c^MLM: machine learning modeling.

^d^SCORAD: Scoring Atopic Dermatitis Index.

^e^NR: not reported.

The definitions for scratching differed widely. The earliest study [26] had the following description for 3 categories of scratching: “light rubbing, touching or scratching” with any part of the body that lasted <5 seconds (light scratching), 5-30 seconds (short bouts of scratching), or >30 seconds (long bouts of scratching). The most detailed definition of scratching [25] found among all of the articles was “any repetitive rubbing or scratching of the body—including through fabric...performed with any part of the body.” In this publication, the definition of time spent scratching (scratching time) was considered to start with the first video frame showing movement consistent with this action and to end with the last contact with the scratched area or if no movement had occurred in that area for at least 3 seconds. Another common method of defining scratch for the purposes of measurement took the form of instructing study participants to perform a willful scratching movement and learning from that recorded action. Among the 29 publications, we found 7 variables commonly specified, even though most publications only specified 1 or 2 of these variables. The variables were (1) properties of the scratching movement (eg, speed, frequency, pressure, intensity), (2) the scratching tool (hand, fingers, other body part), (3) the body part or area scratched, (4) the duration of scratching bout, (5) minimum separation between bouts, (6) scratch outcome to be measured, and (7) the digital sensing product or method used to record or observe scratching (Table 2).

**Table 2:** The most commonly defined and used properties of measured scratching with their definitions and examples.

Property type and name		Definition	Example
**Required**			
	Characteristics of the scratching movement	Aspects of the scratching movement	Speed [52], frequency (in Hz) [32,33,38,47,52], pressure [27,39], force used [35,52], acceleration [42,49], velocity [42], etc. Intensity was defined in 1 article as a composite of pressure force, frequency, and speed [52]. In another article, intensity was defined by the articulation of the body parts (“weak” for fingers only, “moderate” including elbow, “strong” if both elbow and shoulder were moving) [49].
	Scratch Outcome to be Measured	A final outcome that measures the concept of interest (scratching)	Total scratching time [11,28,30], amount of accelerations >0.01 *g* [38], wrist activity in 0-3 Hz range over the first 3 h of sleep [38], total scratch records per “sleeping time” [29]
**Preferred**			
	Scratching tool	The body part used for scratching	Hands, fingers, fingernails, or feet [11,26-28,30,38,39,42,45,49,52]
	Body part or area scratched	The areas or body parts targeted by the scratching action	Head, face, and neck; right upper extremity; left upper extremity; torso, including back; right lower extremity; left lower extremity [25]; facial areas [39,42,43,45]; any part of the body [11,26,28,30,32,47]
	Duration of scratching bout and minimum separation between bouts	Minimal measured duration of scratching; the minimum time between 2 measured scratching bouts. A pause shorter than this minimum time would result in continued measurement of a scratching bout without a pause.	Scratch bout=5 s [11,25,26,28,30,39,45,47,48], 3 s [41,45,49], 2 s [38], 1 s [32]; separation between bouts=3 s [11,25,26,28,30,39,47]
	Method or tool of recording scratch	The methods or tools used for recording scratching or time during which scratching was monitored. In case of DHTs^a^, also sensor type or algorithm used.	Methods: videography [11,25,26,28,32,41,44,46,47,49,52], accelerometry [11,25,27,29,30,32-34,38-40,42,45,47-49], electromyography [36,39], diary [50], survey [32,51]; tools: infrared CCD^b^ camera [11,26,28,47,52], various wearable wrist accelerometer devices [11,25,27,29,30,32-34,38-40,42,45,47,49], acoustic devices [11,38,52]

^a^DHT: digital health technology.

^b^CCD: charged-coupled device.

### Targeted Review of Sleep Period Definitions

Compared with the term “scratch,” the concept of “nocturnal” or, more contextually accurately, “sleep” is more comprehensively researched and well defined. Leveraging the large corpus of existing literature on sleep and existing definitions, we performed a targeted review of the sleep-related search terms describing a period of time one spends with the intention to sleep or in actual sleep, either on its own or together with a specific symptom or condition happening during sleep. The search for secondary symptoms during sleep returned 154 papers (title search) in PubMed. The search for “sleep opportunity” returned 23 papers (title search) in Google Scholar and 127 (title and abstract search) papers in PubMed. Searching for the term “attempted sleep” resulted in 354 papers in Google Scholar and 0 papers in PubMed. We used these papers to find standardized definitions of the period during which sleep occurs. We used the information from the found papers to inform the ontology of sleep.

### Refining the Meaning of “Nocturnal” in the Context of Nocturnal Scratch

We also searched for definitions or clarifications of what the researchers meant by “nocturnal” in the papers that have detailed descriptions of “scratch.” Among the final 29 publications from the scratch search, only 2 had any description of sleep variables and their definitions for nocturnal scratching (Table S1 in Multimedia Appendix 1) [27,29]. Other publications did not specifically define the sleep period during which scratching was measured, despite using methods measuring sleep such as polysomnography.

These 2 studies used identical descriptions for sleep. They defined the sleep start time and sleep end (wake) time. These were defined as when the participants attached the scratch monitor before going to sleep until the time when the participants removed the scratch monitor when they woke up. However, a definition of the measurement period overlapping with participants’ sleep was not provided in more detail or by using standard sleep outcome measure terms, such as “sleep opportunity.” No control method for observing or measuring sleep was used in these studies. Hence, based on the findings in our targeted literature search, we developed ontologies to clarify the terms defining the sleep period for the context of nocturnal scratching, which could be easily confused otherwise.

Our analysis of both scratch and sleep publications brought a conclusion that nocturnal scratch should not be measured during the period of “night” but rather during the period of “sleep.” This is an important recontextualization of the term that has not occurred before. The measurement of scratching within the context of attempted and actual sleep will provide a common understanding of the term, better define the measurement period, and accommodate patients’ needs and behaviors.

### Ontologies and Terminology

**Figure 2:**
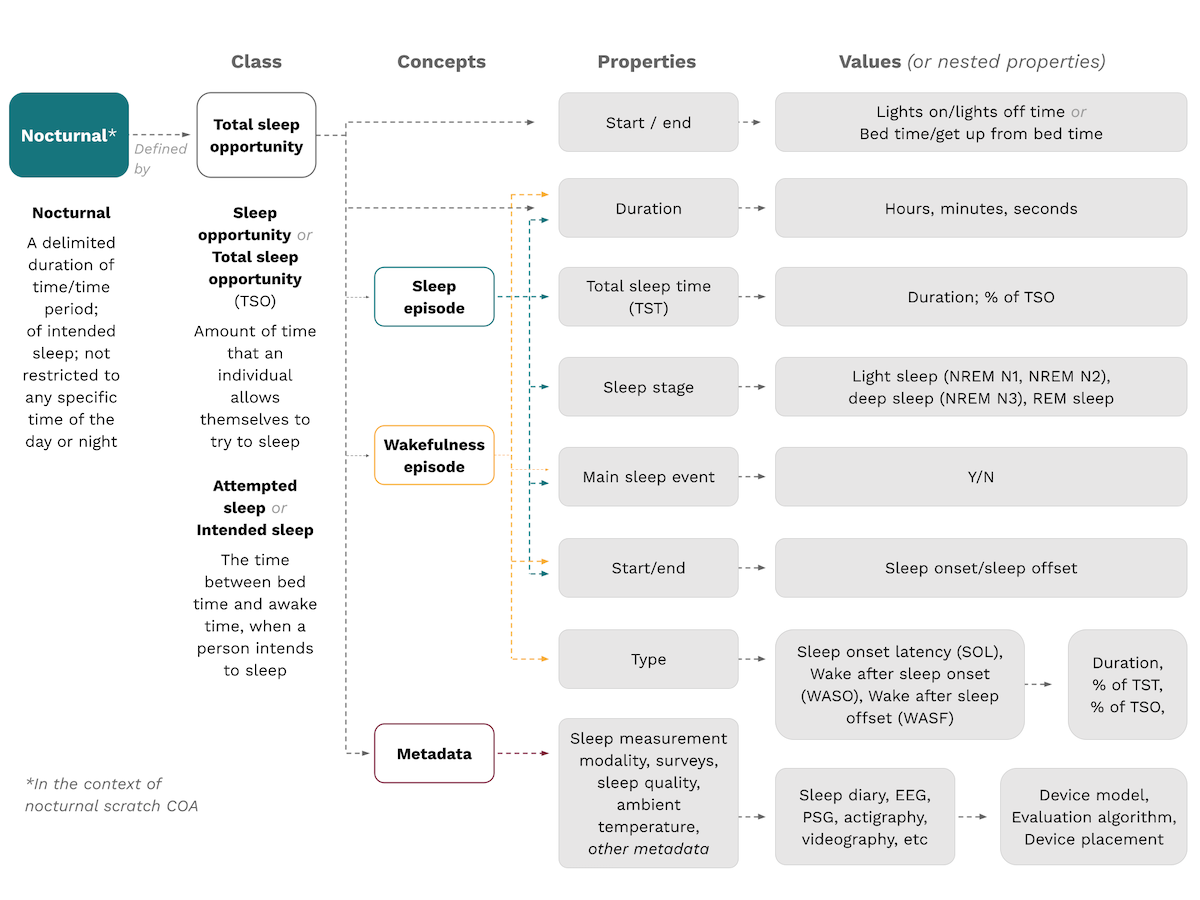
Proposed ontology for digital measurement of sleep. COA: clinical outcome assessment; EEG: electroencephalogram; NREM: non–rapid eye movement; PSG: polysomnography; REM: rapid eye movement.

**Figure 3:**
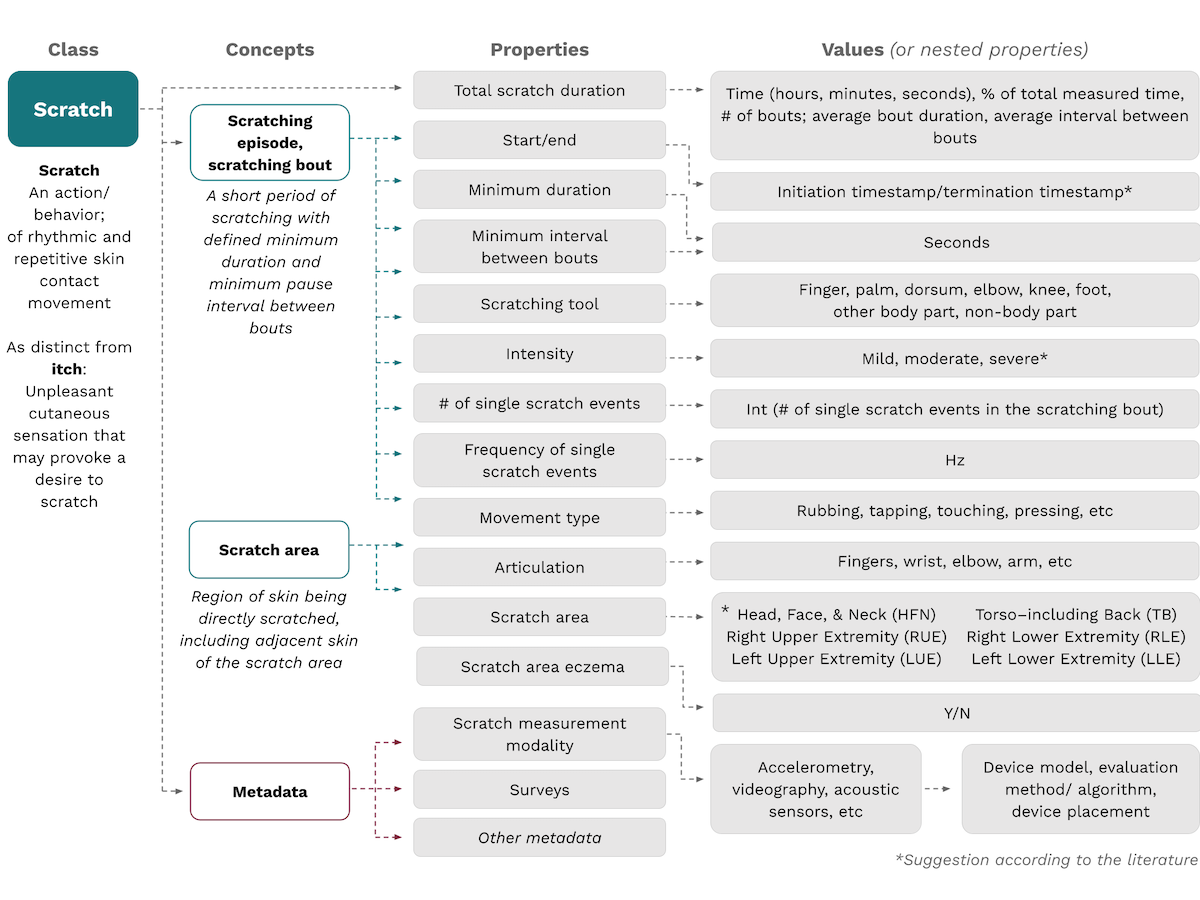
Proposed ontology for digital measurement of dermatitis-related scratching.

To begin the effort toward standardization of definitions and selection of fit-for-purpose outcome measures, we developed ontologies for the digital measurement of sleep scratching (Figures 2 and 3). For “nocturnal” (Figure 2), this resulted in recontextualizing this term as the period of intended and actual sleep. From this, main concepts were defined (sleep episode, wakefulness episode, metadata), with specific properties and values appearing for each concept. As an example, the properties that were measured for the concept “sleep episode” included duration, total sleep time, sleep stage, the main sleep event, and start and end.

For “scratching” (Figure 3), we created an ontology that was structurally identical to that created for sleep. For this term, the concepts were scratching episode, scratch area, and metadata. These concepts were described by the properties of duration, tool used, intensity, number of events, and movement type, with specific values attached to each property.

By deconstructing the terms “sleep/rest/nocturnal” and “scratch” and building ontologies to define constituent concepts and their relationships, we reached our goal of developing a standardized definition of scratch during rest or sleep, otherwise known as nocturnal scratch. This evidence-based and patient-centric definition we propose for nocturnal scratching, or scratching during sleep, is that it is an action of rhythmic and repetitive skin contact movement performed during a delimited time period of intended and actual sleep, not restricted to any specific time of the day or night (Table 3). For further clarity, the concepts underlying this definition can be dissected according to the ontologies in Figures 2 and 3.

**Table 3:** Definition and main outcome measure of nocturnal scratch.

Term	Definition	Main outcome measure
Nocturnal scratch	Action of rhythmic and repetitive skin contact movement performed during a delimited time period of intended and actual sleep, not restricted to any specific time of the day or night	Total scratch time (sum of all recorded scratching bouts) measured during a delimited period of intended and actual sleep within the total sleep opportunity

It is important that a main outcome measure for nocturnal scratching is brought forward in this work. Based on the literature review and developed ontologies, we can propose the main outcome measure of nocturnal scratch: the total scratch time (sum of all recorded scratching bouts) measured during a delimited period of intended and actual sleep within the total sleep opportunity (Table 3). Total scratch time can be also represented as a percentage of the measured sleep period. These definitions of measurement properties and main outcome measures of nocturnal scratching are technology agnostic and can be universally adopted across various sensor technologies or algorithms developed to measure and assess these phenomena.

## Discussion

### Principal Results

In this review of the literature, definitions for scratching-related variables differed widely. These differences—due to varying definitions appropriated for devices, analytical techniques, and study goals—hamper the ability to develop standardized outcome measures to assess and compare digital technologies and to monitor various scratching-related disease states in diverse patient cohorts. In addition, only 2 studies provided any description of the variables used to define sleep, both of which used the same device-specific characteristics. A variety of techniques with inadequate a priori definitions were used to determine the sleep/wake times in the other studies, also hampering cross-study comparisons.

For nocturnal scratching, the term “nocturnal” is itself an exclusionary misnomer. Conventionally, the term describes actions or events that happen at night (eg, nocturnal animals). However, in medicine, this term is often used for the actions or activities occurring during the time of sleep (eg, nocturnal incontinence). Although the term “nocturnal” in the measurement of nocturnal scratching has been historically used and well established, it has not been precisely defined in the context of patients’ sleep. In a world where researchers and drug developers are becoming more focused on patient centricity, the term “nocturnal” has become insufficient, as patients’ sleep can occur during different parts of the day due to various reasons or situations. Examples include shift workers, cross time zone travelers, infants, or people with sleep disorders. As this term is rooted in a period of sleep, using the closest sleep period definition and outcome measure—sleep opportunity—provides an elegant solution to establish the term for the purposes of measurement.

While the term “scratch” is widely understood, a unified definition has never been proposed. A definition discerning it from the sensation of itch is often not provided in research studies or during interactions with patients. Moreover, the literature review showed that different research studies often have different definitions of scratching, its measurement, and its properties.

To aid in a unified understanding, we created ontologies for the digital measurement of scratching during sleep based on our literature review. In the ontologies, the properties and definitions for the main concepts of sleep and scratch, along with their nested properties, present a road map for the development of detailed standardized measurements for use in clinical research and practice. A rich list of properties available to add to assessments will ensure improved specificity of measurement and thus add valuable information about the patient populations.

Per our ontologies, the inclusion of more advanced properties remains to be decided by specific research groups or tool developers. To use sleep as an example, this would include whether rapid eye movement (REM) and non-REM sleep periods are included in the definition; whether wake periods are included in the overall sleep period measured; and whether resting on the bed before falling asleep, resting in bed before waking up, and waking after sleep onset “count” toward the sleep period. These aspects will need to be specified by the researchers prior to every measurement of scratching during sleep, and we recommend incorporating them based on the measurement ability of the tools used. While some of these variables are optional, we believe that several of the properties should be required at all times, including the definition of the sleep period considered and the device or method used to measure sleep—whether polysomnography, actigraphy, wearable smart watches, or observer recordings.

In this work, we propose a unified definition of sleep time scratch, or “nocturnal scratch,” together with a list of concepts and properties allowing for the efficient measurement of this phenomenon. Our goal is to enable better communication and sharing of results between various stakeholders taking part in research in AD and other skin inflammatory conditions. The evidence-based definition of the measurement itself, its properties, and main outcome measures provide everyone with a powerful “dictionary” that can ultimately drive rapid acceptance of novel DHT-based measurements in clinical research by the patients themselves, clinicians, industry, technology developers, regulators, and payers. The definition also provides clear specifications for developing digital products that will no longer need to differentiate themselves based on the varied measurement properties but that can now focus on usability, utility, and other features. As a result, researchers, clinicians, and patients will be able to make better choices across high-quality measurement tools.

Digital biomarkers and digitally measured clinical outcome assessments must be subject to reproducible and meaningful verification and algorithmic and clinical validation [55]. Without meaningful and comprehensive definitions, however, there can be no reliable verification or validation procedures. In addition, researchers can tune the properties (variables mentioned above) to achieve better performance or specificity of information, but that does not necessarily mean better performance and usefulness in the actual application of a digital biomarker. The context of the disease and specifics of the patient population for the measurement will always be important aspects to consider when deliberating the parameters of measurements, as will the value of the measure to the patients themselves [56].

We are hopeful that these definitions and ontologies will become standardized and common through their wide adoption. Even the origination of the idea for this research supports these outlooks, as it was sparked by a precompetitive collaboration [57] between industry and invited experts from academia, clinical space, patient organizations, and regulatory bodies. We encourage all these stakeholders to use the proposed ontologies and terminology in their research and evaluation of evidence on the digital measurement of nocturnal scratch to drive unified advancement in the adoption of DHT-based measurements. We are aware that agreeing on the standard values across all research requires a consensus between technology developers, clinicians, patients, and regulators, which is not easily achievable. However, we believe by the standardization of definitions and properties based on data and evidence, as well as by expert discussions and future collaborations, the goal of introducing a unified measurement will eventually be achieved.

### Limitations

This study has limitations. First, the considerable variations in study design and populations for the studies included in this review prevented the assessment of study quality through established evaluation tools [58]. We thus attempted to quantify the quality of evidence in simpler terms by describing the cohort size and particulars of the measurement instruments and methods. In addition, some relevant studies might have been overlooked in our searches, although we included as many applicable terms as possible in the search strings. Finally, studies with negative results might not have been published at all (due to positive publication bias), possibly skewing our findings.

### Conclusions

Wearable digital technologies and machine learning algorithms show potential in the assessment and monitoring of scratching during sleep. Standardized evidenced-based definitions for variables related to this symptom will aid in evaluations of these tools and their wide adoption in clinical research.

## Appendix

Multimedia Appendix 1 Supplementary tables.

## Abbreviations

AD: atopic dermatitis
DHT: digital health technology
EASI: Eczema Area and Severity Index
IGA: Investigator Global Assessment Scale
POEM: Patient-Oriented Eczema Measure
REM: rapid eye movement
SASSAD: Six Area, Six Sign Atopic Dermatitis Scale
SCORAD: Scoring Atopic Dermatitis Index
