# Supplementary material for: Defining the Digital Measurement of Scratching During Sleep or Nocturnal Scratching: Review of the Literature
Source: J Med Internet Res. 2023 Apr 18;25:e43617. doi: 10.2196/43617 (PMC10155092; doi:10.2196/43617)
Supplement: Multimedia Appendix 1 [file jmir_v25i1e43617_app1.pdf]

Multimedia Appendix 1, Wang et al. Defining Digital Measurement of Scratching During Sleep, or "Nocturnal Scratch": A Review of the Literature

Supplementary Table 1. Search strategies for literature databases - Scratch.

PubMed	("dermatitis"[MeSH Terms] OR "dermatitis"[All Fields] OR ("skin"[All Fields] AND "inflammation"[All Fields]) OR "skin inflammation"[All Fields] OR "inflamed skin"[All Fields] OR Eczema*[All Fields] OR "Prurigo Nodularis"[All Fields] OR "dermal inflammation"[All Fields] OR ("dermal"[All Fields] AND "inflammation"[All Fields]) OR "inflamed dermis"[All Fields] OR "inflammatory skin disease"[All Fields] OR "inflammatory skin diseases"[All Fields] OR "acrodermatitis"[All Fields] OR "intertrigo"[All Fields] OR "neurodermatitis"[All Fields] OR "radiodermatitis"[All Fields] OR "drug eruptions"[All Fields] OR "drug eruption"[All Fields])<br>AND<br>(scratch* [title] OR itch* [title] OR pruritus* [title])
IEEE	"Abstract": "dermatitis*" OR "Abstract": "skin inflammation" OR "Abstract": "inflamed skin" OR "Abstract": "eczema" OR "Abstract": "Prurigo Nodularis" OR "Abstract": "dermal inflammation" OR "Abstract": "inflamed dermis" OR "Abstract": "acrodermatitis" OR "Abstract": "intertrigo" OR "Abstract": "neurodermatitis" OR "Abstract": "radiodermatitis" OR "Abstract": "drug eruption" OR "Abstract": "inflammatory skin disease*"
SCOPUS	(TITLE("itch") OR TITLE("pruritus") TITLE("scratch") OR TITLE("scratching"))<br>AND<br>(TITLE-ABS-KEY("dermatitis") OR TITLE-ABS-KEY("skin inflammation") OR TITLE-ABS-KEY("inflamed skin") OR TITLE-ABS-KEY("eczema") OR TITLE-ABS-KEY("Prurigo Nodularis") OR TITLE-ABS-KEY("dermal inflammation") OR TITLE-ABS-KEY("inflamed dermis") OR TITLE-ABS-KEY("acrodermatitis") OR TITLE-ABS-KEY("intertrigo") OR TITLE-ABS-KEY("neurodermatitis") OR TITLE-ABS-KEY("radiodermatitis") OR TITLE-ABS-KEY("drug eruption") OR TITLE-ABS-KEY("inflammatory skin disease"))
Web of Science	KP =(("dermatitis*" OR "skin inflammation" OR "inflamed skin" OR "eczema" OR "Prurigo Nodularis" OR "dermal inflammation" OR "inflamed dermis" OR "acrodermatitis" OR "intertrigo" OR "neurodermatitis" OR "radiodermatitis" OR "drug eruption" OR "inflammatory skin disease*"))<br>AND<br>TI= (itch OR itching OR scratch OR scratching OR pruritus)

Supplementary Table 2. Search strategies for literature databases - Sleep.

PubMed	("leg cramp*" [Title] OR pain [Title] OR scratch [Title] OR rheumat* [Title] OR epilep* [Title] OR spasm [Title]) AND (nocturnal [Title] OR sleep [Title] OR sleep opportunity [Title] OR nap [Title] OR rest [Title]) AND ("diagnostic manual" [All Fields] OR "diagnostic guide*" [All Fields] OR "review" [All Fields])
PubMed	"sleep opportunity" [tiab]
PubMed	"attempted sleep" [tiab]
Google Scholar	"sleep opportunity" (title search using software "Publish or Perish")
Google Scholar	"attempted sleep" (all fields using software "Publish or Perish")

Supplementary Table 3. Details of studies included in the review - scratch.

**Author (Year)**	**Scratching Behaviour**	**Scratching Tool**	**Scratching Site**	**Scratch Duration**	**Clinical Outcome or Calculated Index**	**Device**	**Comment**	**Sleep Definition/Terminology**	**Detection Method**	**Notes**
Ebata et al. (1996)[1]<br>n = 10	Apparent action of rubbing or scratching to any part of body with a rhythmical movement using hands or fingers, and sometimes feet.	Hands, fingers or feet	Flexible	With more than 3-s separation:<br>1) light rubbing, touching, or scratching lasting <5 s (LR)<br>2) short bouts of scratching with a duration 5–30 s (SS)<br>3) long bouts of scratching with a duration >30 s (LS).	Total Scratching Time (TST), TST% (TST/total recording time)	Infrared video camera			Manual recognition and count through video recording	
Endo et al. (1997)[2]<br>n = 43		Hands	Flexible	>3 changes in pressure at a rate of ≥1/s	- 'Scratch rate'=total time with scratch records other than 0 (min.)/ 'sleeping time' (min)<br>- 'Minute scratch records' (No./min)=total scratch records/'sleeping time' (min)	Scratch monitor (SM)		- **'Asleep':** first time SM records 0 for ≥10 min, beginning of 0<br>- **'Awake':** time when scratch records other than 0 begin to just before removal of SM<br>- **'Sleeping time'** (min): time from 'asleep' to 'awake'<br>- **'Pre-asleep time'** (min): time from attachment of SM to 'asleep'<br>- **3 phases of sleeping period** (early, mid, late period): 3 equal parts of sleeping time from 'asleep' to 'awake'<br>- **'Sound sleeping state'** (min)**:** when SM records 0 continuously for >10 min<br>- **'Sound sleep rate':** sum of time in 'sound sleeping state'/ 'sleeping time'	Pressure changes in scratch monitor	

Author (Year)	Scratching Behaviour	Scratching Tool	Scratching Site	Scratch Duration	Clinical Outcome or Calculated Index	Device	Comment	Sleep Definition/Terminology	Detection Method	Notes
								- **'Awake bouts'**: when a series of scratch records other than 0 contains >50 or a number other than 0 continues for >5 min<br>- **'Hourly awake number'** (no/h): number of 'awake bouts'/ 'sleeping time'<br>- **'Awake rate:** sum of time in 'awake bouts' (min) /'sleeping time' (min)		
Ebata et al. (1999)[3]<br>n = 35	Apparent action of rubbing or scratching on any part of body with a rhythmical movement using hands or fingers, and sometimes feet.	Hands, fingers, or feet	Flexible	5 s with at least 3-s separation	Total Scratching Time (TST), TST% (TST/total recording time)	Infrared CCD camera			Manual recognition and count through video recording	
Endo et al. (1999)[4]<br>n = 40		Hands	Flexible	>3 changes in pressure at a rate of ≥1/s	- 'Scratch rate'=total time with scratch records other than 0 (min.)/ 'sleeping time' (min)<br>- 'Minute scratch records' (No./min)=total scratch records/'sleeping time' (min)	Scratch monitor (SM)	Also included great terminology of sleep related metrics	- **'Asleep':** first time SM records 0 for ≥10 min, beginning of 0<br>- **'Awake':** time when scratch records other than 0 begin to just before removal of SM<br>- **'Sleeping time'** (min): time from 'asleep' to 'awake'<br>- **'Pre-asleep time'** (min): time from attachment of SM to 'asleep'<br>- **3 phases of sleeping period** (early, mid, late period): 3 equal parts of sleeping	Pressure changes in scratch monitor	

Author (Year)	Scratching Behaviour	Scratching Tool	Scratching Site	Scratch Duration	Clinical Outcome or Calculated Index	Device	Comment	Sleep Definition/Terminology	Detection Method	Notes
								time from 'asleep' to 'awake'<br>- **'Sound sleeping state'** (min)**:** when SM records 0 continuously for >10 min<br>- **'Sound sleep rate':** sum of time in 'sound sleeping state'/ 'sleeping time'<br>- **'Awake bouts'**: when a series of scratch records other than 0 contains >50 or a number other than 0 continues for >5 min<br>- **'Hourly awake number'** (no/h): number of 'awake bouts'/ 'sleeping time'<br>- **'Awake rate:** sum of time in 'awake bouts' (min) /'sleeping time' (min)		
Ebata et al. (2001)[5]<br>n = 21, 34	Apparent action of rubbing or scratching on any part of body with a rhythmical movement using hands or fingers, and sometimes feet.	Hands, fingers, or feet	Flexible	5 s with at least 3-s separation	Total scratching time (at night)	ActiTrac®			Piezoceramic sensor to record physical motion in 2 planes by acceleration signals, which is sampled at a rate of 40x/s and is digitally integrated to quantify all activity under signal curve. This information is converted to a reference scale of data counts (0–250), which correspond to precisely calibrated 0–75*10^3g units of acceleration.	

Author (Year)	Scratching Behaviour	Scratching Tool	Scratching Site	Scratch Duration	Clinical Outcome or Calculated Index	Device	Comment	Sleep Definition/Terminology	Detection Method	Notes
Munday et al. (2002)[6] n = 155					5 items (erythema, excoriation, dryness, lichenification, exudation and crusting) were scored using a digital VAS (Innovaderm)	Surveys				
Benjamin et al. (2004)[7] n = 21	Activity was classified operationally into 4 categories: sleeping; scratching; restless movement; and movement under covers. Time spent in each activity was recorded in minutes. Scratching was defined to include rhythmic movements, judged by eye, greater than 1 Hz without accompanying movements. By contrast, restlessness—such as frequent turning over in bed, slow rubbing of face on pillow, or writhing movements of limbs—could include short periods of scratch (1–2 s) as long as these latter movements were interspersed with other non-scratch activities. Thus, whereas scratching activity (in absence of restlessness) could be usefully demarcated, short periods of scratching interspersed with restlessness could not be separated, and were summarized as restlessness. Movement under covers refers to obvious movement that could not be assessed definitely as either belonging to restlessness or scratching.<br>First, as an objective correlate of itch (with itch commonly being defined as that which produces desire to scratch). S, scratch, being result of itch, may, through local damage to skin, exacerbate primary cause of itch. It is, to use an analogy from economics, a "multiplier" (of pathology).	Flexible	Flexible	2 s (accelerometer)<br>1 or 2 s (video)	Time spent scratching, restless, moving under covers, and apparently sleeping (from the total recorded time)	Piezoelectric accelerometer<br>video recording			Piezoelectric accelerometer that records integration of intensity, amount, and duration of stimuli in all 3 dimensions.	Subsequent studies have built on these original approaches and have used various methods to assess scratch activity, including movement transducers attached to bed legs, measurement of forearm muscle potentials, self-winding watches used as accelerometers worn on limb, pressure-sensitive meters on wrist, electromagnetic movement detection, paper strain gauges applied to wrist, and piezoelectric devices applied to fingernails. Videotaping of subjects has also recently been reported...
Hon et al. (2006)[8] n = 39	Wrist activities between 1 and 3 Hz				1-3 Hz wrist activity in the first 3 hours of sleeping	DigiTrac monitor			Wrist activities were expressed in units of average value of acceleration (g/min)	See "Pathophysiology of nocturnal scratching in childhood atopic dermatitis: role of brain-derived neurotrophic factor and substance P"
Hon et al. (2007)[9] n = 28	Limb motion 1–3 Hz	Limb			Wrist activity between 1 and 3 Hz for first 3 h of sleep	DigiTrac monitor on their			DigiTrac was found to provide essential data on a wide spectrum of frequencies of wrist	

Author (Year)	Scratching Behaviour	Scratching Tool	Scratching Site	Scratch Duration	Clinical Outcome or Calculated Index	Device	Comment	Sleep Definition/Terminology	Detection Method	Notes
						dominant wrist			movements and quantity of movements in terms of acceleration or g values.	
Ishiuji et al. (2008)[10] n = 26	Scratching was accomplished by study personnel by repetitively moving a cytology brush over ventral forearm 3 cm distal to iontophoresis site. Uniformity was controlled by applying sufficient pressure to bend skin-facing brush bristles so that brush handle touched skin surface. Bending force of cytology brush was equivalent to ~29g on a digital scale. Cytology brush contains ~1000 individual bristles, and the diameter of brush was 7.5 mm. The same member of research team applied the cytology brush for all subjects. Time course of scratching was identical to that of thermal stimulation. Each examinee underwent a training session in which study personnel applied cytology brush.	Medi-Pak 7" cytology brush 24–2199; General Medical Corporation, Richmond, VA USA			Perceived itch intensity screened with VAS (0-100).		Scratching was performed through an electronic device		Simulation: itch was evoked by medicine and scratch was performed by electronic device. Not natural stimulus and response.	
Bender et al. (2008)[11] n = 20	Participants were videotaped during polysomnography. Sleep technician observed and recorded each period in which scratching occurred. A scratching event was recorded as such when a burst of electromyographic activity of ≥3 s was accompanied by visible scratching motion.			3 s	Scratching index	PSG, electromyograph	Couldn't find clear definition or description of scratch		A scratching event was recorded as such when a burst of electromyographic activity of ≥3 s was accompanied by visible scratching motion.	What's unit for scratch index and how is it calculated from videotape?
Tran et al. (2010)[12] n = 45	Subjects underwent artificial scratching over area on forearm 3 cm distal to edge of area of histamine iontophoresis for 2 min. Scratching was accomplished by study personnel repetitively moving a cytology brush over ventral forearm. Uniformity was controlled by applying sufficient pressure to bend skin-facing brush bristles so that brush handle touched skin surface, as described previously. The same member of the research team applied the cytology brush for all subjects. Itch ratings were obtained using a numerical scale from 0–10, 1 min after scratching stimulus was applied.	Medi-Pak 7" cytology brush 24–2199; General Medical Corporation, Richmond, VA USA	Forearm	2 minutes	Itch-score in 0–10	Survey	Scratching was performed through an electronic device		Simulation: itch was evoked by medicine and scratch was performed by electronic device. Not natural stimulus and response.	
Petersen et al. (2013)[13] n = 12	[From referenced papers] movement with frequency of 1–2 Hz.	Hands or fingers		[From reviewed papers] segmenting data into 2-s epochs	Activity in 0–3 Hz range over first 3 h of sleep number of accelerations >0.01g	Triaxial accelerometry (PAM-RL, Philips Respironics, OR, USA),			Acceleration, by using positions on each axes	

Author (Year)	Scratching Behaviour	Scratching Tool	Scratching Site	Scratch Duration	Clinical Outcome or Calculated Index	Device	Comment	Sleep Definition/Terminology	Detection Method	Notes
						Acoustic and others				
Kurihara et al. (2013)[14] n = 10	Subject was instructed to scratch right cheek 20 times, because patients with atopic dermatitis most often scratch head area as a characteristic pattern. He then returned his arm to starting position. After a 5-s pause, subject once again scratched his cheek 20 times in a row. This set of scratching motions was repeated 35 times.	Dominant hand	Cheek or head area	5 s	Total scratching time % After obtaining all data, clinician also judged scratching time by visual observation of signal waveform.		Scratching movement characterized by experimentation and recording		Changes in myogenic potential of forearm were measured via EMG sensor. Pressure changes on back of hand: a ceramic sheet; Sounds from scratching: microphone fixed to back of hand with surgical tape, output data transmitted through an amplifier and A/D-converted at a sampling interval of 1 ms and scale range of ±10 V using a data logger. Expansion and contraction of fingers: strain gauge (V) Acceleration of forearm (G): triaxial accelerometer embedded in EMG sensor. A/D-converted at a sampling frequency of 204 Hz. Angular velocity of forearm: triaxial gyro sensor.	See above
Price et al. (2014)[15] n = n/a	Apparent action of rubbing or scratching to any part of body with a rhythmical movement using hands or fingers, and sometimes feet.	Hands, fingers, or feet	Flexible	5 s with at least 3-s separation	- Total Scratching Time - SCORAD score - ratio of TST divided by total recording time (TST%)	- Infrared video - Actigraphy - Acoustic device			Manual recognition and count through video recording	Review article
Yamanaka et al. (2015)[16] n = 20					Ratio of scratching duration to sleep period time (SPT), abbreviated as	Wristwatch Electroencephalogram			Wristwatch-type scratching counting system detecting small scratching sounds from fingertip at wrist	

Author (Year)	Scratching Behaviour	Scratching Tool	Scratching Site	Scratch Duration	Clinical Outcome or Calculated Index	Device	Comment	Sleep Definition/Terminology	Detection Method	Notes
					scratching ratio, calculated every 10 min					
Schut et al. (2015)[17] n = 48	Any movement that included rubbing				Induced scratching was determined by subtracting number of scratch movements occurring during viewing of control video from those occurring during viewing of experimental video.	Video and questionnaire				Itch was induced by a non-invasive method, which might work through classical conditioning processes and uses (audio-) visual material.
Lee et al. (2015)[18] n = 3	Simulated scratch and non-scratch movements (e.g., irregular wrist movements) while lying down. Authors extracted distance moved, velocity of wrist movements, and number of scratching bouts. Because scratching is a periodic movement, they regarded consecutive changes of direction of wrist movements as number of scratching. They observed that people may use their wrists and fingers to scratch, but often they use only their fingers. Although scratching with fingers without significantly moving the wrist causes a tiny amount of acceleration, the acceleration pattern for scratching with wrist movement was identical, showing that separate definitions/characterizations of scratch for wrist scratching vs. finger scratching are not needed.	Wrist/fingers	Mostly facial areas (cheek, nose)		Accuracy of detecting scratch vs. non-scratch movements (note: not a clinical, but rather technology outcome)	Accelerometry	Attempt to characterize scratching movement through experimentation. Smartwatch has issue of noise, which may affect data collection.		Accelerometer has 3 axes, each in a certain direction. Gyroscope and magnetometer sensors are used in 3D modelling of wrist movements for detecting spots scratched, which remains as a future work.	
Kurihara et al. (2015)[19] n = nr	Subject moves his right hand to his right cheek, and uses his fingers to scratch his cheek 20 times and then returns to starting position with his arm at his side on bed. Scratching 1 time was 1 scratching stroke.	Right hand, fingers	Right cheek	~2.5 s of moving arms (arm up) + 5 s of scratching (20 times) + ~2.5 s of moving arms (arm down)<br>Time of 2–7.8 s was judged as a scratching period by visual observation. During this period, output signal from strain gauge and piezoceramic sensors showed a cyclic	Strain gauge to measure degree of finger bending, which reflects scratching motion; the closer the piezoceramic sensor is to scratching point, the larger output signal is.	Piezoceramic sensor between 2 metal plates	Characterization of scratching by recording self-driven scratching act		Amplitude (V), by vibrations produced by scratching, vibration is detected by piezoceramic sensors on different locations of bed<br>Degree of finger bending, measured by strain gauge (variable resistance) on finger	

Author (Year)	Scratching Behaviour	Scratching Tool	Scratching Site	Scratch Duration	Clinical Outcome or Calculated Index	Device	Comment	Sleep Definition/Terminology	Detection Method	Notes
				change after scratching motion.						
Camfferman (2016)[20]<br>n = 33	Scratching was deemed not present when there were no excoriations.<br>- Mild scratching described as scant, superficial excoriations.<br>- Moderate scratching described as many superficial and/or some deeper excoriations.<br>- Severe scratching described as diffuse, extensive superficial and/or many deep excoriations.				SCORAD-scratch	PSG			Nocturnal video of each participant's sleep was recorded in conjunction with overnight PSG and later reviewed by a sleep technician for scratching events.	
Kaburagi et al. (2017)[21]<br>n = 12	Subject scratched his right cheek 20 times using fingers of his right hand. He then returned his arm to starting position. After a break of 5 s, subject once again scratched his cheek 20 times in a row. This set of scratching motion was repeated 35 times.	Right hand fingers	Cheek	5 s	Ts : Scratching period<br>Tm : Total measurement time<br>N : Number of scratching periods<br>Total sleep time %		Scratching movement characterized by experimentation and recording		Strain gauge was fixed along index finger to measure degree of bending of finger; accelerometer and a gyro sensor were fixed onto forearm to measure acceleration and angular velocity of forearm	See “Development of vibration sensing system with wide dynamic range: monitoring of scratching and turning-over motions during sleep”
Bartels et al. (2018)[22]<br>n = 97	Any skin contact movement that could reduce itch, e.g. typical scratching using fingernails, picking with fingernails, or rubbing, while not taking into account touching	Fingernails or rubbing	Any part of skin	2 minutes(?)	Frequency and duration of scratching episodes during the measured phase (time period)	Video camera			Manual recognition and count through video recording	Simulation
Moreau et al. (2018)[23]<br>n = 24	Apparent action of rubbing or scratching to any part of body with a rhythmical movement using hands or fingers.	Hands or fingers	Flexible	Each bout of scratching must last longer than 5 s. Scratching bouts with intervals between them of less than 3 s were considered to be a single bout. Scratching duration has threshold of 1.75 s, duration <2 s scored by video (in total about 1.6% of 1-s epochs) were removed. Video and actigraphy scratching events were compared on a 1-s epoch basis.	F1 score of the algorithm assessing scratch/non-scratch movements (note: not a clinical, but rather technology outcome)	Wrist actigraphy (GeneActiv, Activinsights Ltd.) sleep laboratory with IR video.			2 accelerometer devices + video	Gold standard scratching assessment method is by scoring video recordings of patients, but this method is impractical for trials or routine monitoring because of its cost due to laborious scoring, invasion of patient's privacy, and possibility of visual obstructions. Consequently, current clinical standard for assessing scratching behaviour in trials is use of patient-reported

Author (Year)	Scratching Behaviour	Scratching Tool	Scratching Site	Scratch Duration	Clinical Outcome or Calculated Index	Device	Comment	Sleep Definition/Terminology	Detection Method	Notes
										outcomes (e.g. questionnaires) but these measures are imprecise and often do not agree well with objective measures.<br><br>Investigators have used various objective measures to assess scratching—measurement of bed movements, finger flexation, sound detection, actigraphic assessment of sleep quality and quantity, actigraphic measures of motion, and attempts to actigraphically identify scratching events—but none have shown sufficient accuracy and practicality in real-world situations to be accepted for routine use in trials of therapies for pruritus.
Sanders et al. (2019)[24]<br>n = n/a	[Neurological] Scratching activates spinal interneurons that inhibit itch-sensitive neurons, suppressing transmission of itch signals to brain. Scratching an itch also deactivates amygdala, calming our negative emotions. In this rare case, pain becomes something that we actually seek out in order to experience relief from itch.<br>Pleasure of scratching is correlated with activity in major reward centres of brain, such as ventral tegmental area and nucleus accumbens.<br>Therefore, in scratching an itch, we experience 2 rewards: relief of itch (negative reinforcement) and pleasure (positive reinforcement).									Viewpoint article
Ikoma et al. (2019)[25]<br>n = 236	Predefined acceleration pattern that algorithm is looking for, similar to a sine wave.<br>Recording and experimental approach of this project: Each subject wore 2 identical smartwatches (Apple Watch Sport 38 mm, Apple Inc., USA), in which app	Wrist/hand fingers (weak motion)<br>elbow (moderate motion)		Algorithm considers scratching to start when change in acceleration in any of 3 dimensions	Scratching duration per hour (note: the results are not clinical, but rather	Video; accelerometer built into a smart			changes in acceleration data on x, y, and z axis with algorithm for analysis	

Author (Year)	Scratching Behaviour	Scratching Tool	Scratching Site	Scratch Duration	Clinical Outcome or Calculated Index	Device	Comment	Sleep Definition/Terminology	Detection Method	Notes
	was installed, with 1 watch on each wrist. Evaluator, who was blinded to smartwatch data, watched video of subjects and recorded timing and duration of all body motions during sleep. If motions were judged as scratching by evaluator, they were recorded together with information on which arm was used and categorized into 3 grades depending on intensity of scratching motions: "weak" if only fingers were used to scratch without visible movement of elbow, "moderate" if elbow visibly moved, and "strong" if both of elbow and shoulder visibly moved.	elbow and shoulder (strong motion)		continuously meets all of following conditions for ≥3 s, and considers scratching to end when ≥1 condition is no longer met during a predetermined minimum number of consecutive periods in same dimension:<br>- length of a period (TX) does not exceed a predetermined upper limit. A period is defined as the time between when acceleration measured by actigraph sensor first is 0 and when it is again 0<br>- the differences in successive TXs do not exceed a predetermined upper limit;<br>- maximum absolute value of acceleration during TX (MX) is not less than a predetermined lower limit;<br>- the difference in successive MXs do not exceed a predetermined upper limit.<br>- error rate (ER) does not exceed a predetermined upper limit, with ER defined as percentage of TXs that meet none of the above conditions.<br>Scratching motions with intervals of <3 s were considered as single scratching episodes.	technology outcomes)	watch (Apple)				

Author (Year)	Scratching Behaviour	Scratching Tool	Scratching Site	Scratch Duration	Clinical Outcome or Calculated Index	Device	Comment	Sleep Definition/Terminology	Detection Method	Notes
Kamath et al. (2020)[26]<br>n = 37					Clinician Scratch Scale also assessed by investigator at baseline. Scale based on clinician observation or physical evidence of scratching, e.g. excoriations. Scored as 0–4, with 4 representing worst scratching.	Diary/ Questionnaire				
Mahadevan et al. (2021)[27]<br>n = 33	- Any repetitive rubbing or scratching of the body (including through fabric) performed with hand or any part of upper limb (from Ebata et al., [2001])<br>- Initiation Frame: First contact of any part of hand – including fingers, nails, palm, and/or dorsum – with scratch area, continuous with scratching behaviour.<br>- Termination Frame: Last contact of any part of hand – including fingers, nails, palm, and/or dorsum – with scratch area, or last visible movement of hand if there is a ≥3-s pause in scratching with hand still in contact with scratching area.<br>**Scratch behaviours resulting from non-hand scratching – Any repetitive rubbing or scratching of body – including through fabric – performed with any part of body other than upper limb (1) [Length may be discussed.]**<br>- Initiation Frame: First contact of scratching body part with scratch area, continuous with scratching behaviour.<br>- Termination Frame: Last contact of scratching body part with scratch area, or its last visible movement if there is a ≥3-s pause in scratching while it is still in contact with scratching area.<br>**Restless behaviours classified as non-scratch behaviour – Any repetitive, generalized movements of body without repetitive motion that would demonstrate scratching activity or that cannot be directly seen due to obstruction (e.g. by a blanket/sheet, by Subject's body, etc.).**<br>- Initiation Frame: First visible movement of repetitive generalized movement.	Flexible	Flexible	5 s with 3 s separation	Total Scratch Events, Toal Scratch Duration	Wearable			Triaxial accelerometer (unit: g), ambient light sensor (unit: Lux), near-body temperature sensor (unit: Celsius), high-pass filter formula	

Author (Year)	Scratching Behaviour	Scratching Tool	Scratching Site	Scratch Duration	Clinical Outcome or Calculated Index	Device	Comment	Sleep Definition/Terminology	Detection Method	Notes
	- Termination Frame: Last visible movement of repetitive generalized movement<br>**Variations**<br>Hand Scratch and Scratch behaviours resulting from non-hand scratching will have several variants meant to capture expected variations in behaviour (e.g. “Hand Scratch, Right Upper Extremity, Mild Intensity”). Each variation will use same Initiation/Termination Frame criteria as general annotation, but will require rater to recognize characteristics of behaviour to select correct variant. Scratch Area is region of skin being directly scratched, and includes adjacent skin of scratch area. Scratching behaviour refers to movement of scratching body part over scratch area.<br>***Both Hand Scratch and Other Scratch annotations will have following variations:***<br>**Scratching Area Location**<br>- Head, face, and neck (HFN)<br>- Right upper extremity (RUE)<br>- Left upper extremity (LUE)<br>- Torso–including back (TB)<br>- Right lower extremity (RLE)<br>- Left lower extremity (LLE)<br>**Scratch will have following variations: - Scratching Intensity**<br>- Mild–below elbow movement only (1)<br>- Moderate–includes elbow movement (2)<br>- Severe–whole limb movement (3)									
Hong et al. (2021)[28]<br>n = 399	Severity of excoriations correlated best with Numerical Rating Scale (NRS)–worst itch (Spearman correlation, ρ=0.50), followed by a Patient-Reported Outcome Measurement Information System Itch Questionnaire–scratching behaviour T score (ρ=0.48), NRS–average itch (ρ=0.41), relative frequency of itch (ρ=0.36), and frequency of itch from eczema (ρ=0.29, all *P*<0.001). Scratching severity showed good reliability (intraclass correlation coefficient range, 0.62–0.69). Overall, 30.6% and 5.5% had moderate (2) or severe (3) SCORAD-scratch scores. Among patients with baseline moderate or severe SCORAD-scratch scores, 18.9% and 13.6% still had moderate or severe scores during follow-up. In repeated-measures regression models, persistent SCORAD-scratch scores were associated with baseline severity of excoriations (adjusted β [95% confidence interval] = 0.51 [0.37–0.65]), Medicaid insurance (−0.35 [−0.65 – −0.04]), and Eczema Area and Severity Index scores (0.03 [0.02–0.04]).				Survey scores					

Author (Year)	Scratching Behaviour	Scratching Tool	Scratching Site	Scratch Duration	Clinical Outcome or Calculated Index	Device	Comment	Sleep Definition/Terminology	Detection Method	Notes
Chun et al. (2021)[29]<br>n = 21	Scratching varies with individuals in response to both internal and external stimuli; can occur via articulation of elbow, wrist, or fingers alone. In mode that engages entire upper arm, fingers press against surface and remain stationary, where movement of forearm articulating at elbow joint drives scratching action. Finger scratching mode involves mainly local movements of fingers, without substantial forearm or wrist motion. Mixtures of these modes are also possible. Depending on location of scratching and degree of itch, individuals interchangeably use different modes of scratching. For any type of scratching action, intensity is also important. Force at point of contact determines friction between fingertips and surface and can thus be considered, along with frequency and speed, as a metric of intensity of a scratching event.	Hand, fingers			Accuracy of detecting scratch vs. non-scratch movements (note: not a clinical, but rather technology outcome)	Video, observer<br><br>ADvanced Acousto-Mechanic strapped to Apple Watch 4			Accelerations measured in a direction perpendicular to surface of skin	

CCD = charged-coupled device; EMG = electromyography; nr = not reported; PSG = polysomnography; VAS =- visual analogue scale.

References

1. Ebata T, Aizawa H, Kamide, R. An infrared video camera system to observe nocturnal scratching in atopic dermatitis patients. J Dermatol 1996 Mar;23(3):153-155. doi: 10.1111/j.1346-8138.1996.tb03990.x
2. Endo K, Sumitsuji H, Fukuzumi T, Adachi J, Aoki T. Evaluation of scratch movements by a new scratch-monitor to analyze nocturnal itching in atopic dermatitis. Acta Derm Venereol 1997 Nov;77(6):432-435. doi: 10.2340/0001555577432435
3. Ebata T, Aizawa H, Kamide R, Niimura M. The characteristics of nocturnal scratching in adults with atopic dermatitis. Br J Dermatol 1999 Jul;141(1):82-86. doi: 10.1046/j.1365-2133.1999.02924.x
4. Endo K, Sano H, Fukuzume T, Adachi J, Aoki T. Objective scratch monitor evaluation of the effect of an antihistamine on nocturnal scratching in atopic dermatitis. J Dermatol Sci 1999 Dec;22(1):54-61. doi: 10.1046/j.1365-2133.1999.02924.x
5. Ebata T, Iwasaki S, Kamide R, Niimura M. Use of a wrist activity monitor for the measurement of nocturnal scratching in patients with atopic dermatitis. Br J Dermatol 2001 Feb;144(2):305-309. doi: 10.1046/j.1365-2133.2001.04019.x
6. Munday J, Bloomfield R, Goldman M, Robey H, Kitowska GJ, Gwiezdziski Z, et al. Chlorpheniramine is no more effective than placebo in relieving the symptoms of childhood atopic dermatitis with a nocturnal itching and scratching component. Dermatology 2002;205(1):40-45. doi: 10.1159/000063138
7. Benjamin K, Waterston K, Russell M, Schofield O, Diffey B, Rees JL. The development of an objective method for measuring scratch in children with atopic dermatitis suitable for clinical use. J Am Acad Dermatol 2004 Jan;50(1):33-40. doi: 10.1016/s0190-9622(03)02480-0
8. Hon K-LE, Lam M-CA, Leung T-F, Kam W-YC, Lee K-CK, Li M-CA, et al. Nocturnal wrist movements are correlated with objective clinical scores and plasma chemokine levels in children with atopic dermatitis. Br J Dermatol 2006 Apr;154(4):629-635. doi: 10.1111/j.1365-2133.2006.07213.x
9. Hon K-LE, Lam M-CA, Wong K-Y, Leung T-F, Ng P-C. Pathophysiology of nocturnal scratching in childhood atopic dermatitis: the role of brain-derived neurotrophic factor and substance P. Br J Dermatol 2007 Nov;157(5):922-925. doi: 10.1111/j.1365-2133.2007.08149.x
10. Ishiuji Y, Coghill RC, Patel TS, Dawn A, Fountain J, Oshiro Y, et al. Repetitive scratching and noxious heat do not inhibit histamine-induced itch in atopic dermatitis. Br J Dermatol 2008 Jan;158(1):78-83. doi: 10.1111/j.1365-2133.2007.08281.x
11. Bender BG, Ballard R, Canono B, Murphy JR, Leung DYM. Disease severity, scratching, and sleep quality in patients with atopic dermatitis. J Am Acad Dermatol 2008 Mar;58(3):415-420. doi: 10.1016/j.jaad.2007.10.010
12. Tran BW, Papoiu AD, Russoniello CV, et al. Effect of itch, scratching and mental stress on autonomic nervous system function in atopic dermatitis. Acta Derm Venereol 2010 Jul;90(4):354-361. doi: 10.2340/00015555-0890
13. Petersen J, Austin D, Sack R, Hayes TL. Actigraphy-based scratch detection using logistic regression. IEEE J Biomed Health Inform 2013 Mar;17(2):277-283. doi: 10.1109/TITB.2012.2204761
14. Kurihara Y, Kaburagi T, Watanabe K. Development of a non-contact sensing method for scratching activity measurement. IEEE Sensors J 2013 May 20;13:3325-3330. doi: 10.1109/JSEN.2013.2264283
15. Price A, Cohen DE. Assessment of pruritus in patients with psoriasis and atopic dermatitis: subjective and objective tools. Dermatitis 2014 Nov-Dec;25(6):334-344. doi: 10.1097/DER.0000000000000077
16. Yamanaka K, Motomura E, Noro Y, Umeda K, Morikawa T, Umeda-Togami K, et al. Olopatadine, a non-sedating H1 antihistamine, decreases the nocturnal scratching without affecting sleep quality in atopic dermatitis. Exper Dermatol 2015 Mar;24(3):227-229. doi: 10.1111/exd.12630
17. Schut C, Muhl S, Reinisch K, Claßen A, Jäger R, Gieler U, et al. Agreeableness and self-consciousness as predictors of induced scratching and itch in patients with psoriasis. Int J Behav Med 2015 Dec;22(6):726-734. doi: 10.1007/s12529-015-9471-5
18. Lee J, Cho D-K, Song S, Kim SH, Im E, Kim J. Mobile system design for scratch recognition. CHI EA '15: Proceedings of the 33rd Annual ACM Conference Extended Abstracts on Human Factors in Computing Systems 2015 Apr:1567-1572. doi: 10.1145/2702613.2732820
19. Kurihara Y, Kaburagi T, Watanabe K, Tanaka H. Development of vibration sensing system with wide dynamic range: monitoring of scratching and turning-over motions during sleep. Artific Life Robotics 2015 Nov;20:372-378. doi: 10.1007/s10015-015-0241-x
20. Camfferman D, Short MA, Kennedy JD, Gold M, Kohler M, Lushington K. Thermoregulation, scratch, itch and sleep deficits in children with eczema. Sleep Medicine 2016 Sep;25:145-150. doi: 10.1016/j.sleep.2016.06.011
21. Kaburagi T, Kurihara Y. Algorithm for estimation of scratching time. IEEE Sensors J 2017 Jan;17:2198-2204. doi: 10.1109/JSEN.2017.2658949
22. Bartels DJP, van Laarhoven AIM, van de Kerkhof PCM, Evers AWM. Nocebo effects and scratching behaviour on itch. Acta Derm Venereol 2018 Nov 5;98(10):943-950. doi: 10.2340/00015555-2979
23. Moreau A, Anderer P, Ross M, Cerny A, Almazan TH, Peterson B. Detection of nocturnal scratching movements in patients with atopic dermatitis using accelerometers and recurrent neural networks. IEEE J Biomed Health Inform 2018 Jul;22(4):1011-1018. doi: 10.1109/JBHI.2017.2710798
24. Sanders KM, Fast K, Yosipovitch G. Why we scratch: function and dysfunction. Exper Dermatol 2019 Dec;28(12):1482-1484. doi: 10.1111/exd.13977
25. Ikoma A, Ebata T, Chantalat L, Takemura K, Mizzi F, Poncet M, et al. Measurement of nocturnal scratching in patients with pruritus using a smartwatch: initial clinical studies with the itch tracker app. Acta Derm Venereol 2019 Mar 1;99(3):268-273. doi: 10.2340/00015555-3105
26. Kamath BM, Spino C, McLain R, Magee JC, Fredericks EM, Setchell KD, et al. Unraveling the relationship between itching, scratch scales, and biomarkers in children with Alagille Syndrome. Hepatol Comm 2020 May 26;4(7):1012-1018. doi: 10.1002/hep4.1522

27. Mahadevan N, Christakis Y, Di J, Bruno J, Zhang Y, Dorsey ER, et al. Development of digital measures for nighttime scratch and sleep using wrist-worn wearable devices. npj Digit Med 2021 Mar 3;4(1):42. doi: 10.1038/s41746-021-00402-x
28. Hong MR, Lei D, Yousaf M, Chavda R, Gabriel S, Janmohamed SR, et al. Reliability and longitudinal course of itch/scratch severity in adults with atopic dermatitis. Dermatitis 2021 Oct 1;32(1S):S28-S32. doi: 10.1097/DER.0000000000000716
29. Chun KS, Kang YJ, Lee JY, Nguyen M, Lee B, Lee R, et al. A skin-conformable wireless sensor to objectively quantify symptoms of pruritus. Sci Adv 2021 Apr 30;7(18):eabf9405. doi: 10.1126/sciadv.abf9405
